# Lipoic Acid Treatment after Brain Injury: Study of the Glial Reaction

**DOI:** 10.1155/2013/521939

**Published:** 2013-11-05

**Authors:** Brenda Rocamonde, Sara Paradells, Carlos Barcia, Angeles Garcia Esparza, José Miguel Soria

**Affiliations:** ^1^Facultad Ciencias de la Salud, Universidad CEU Cardenal Herrera, Moncada, 46313 Valencia, Spain, Spain; ^2^Institut de Neurociències, Facultad de Medicina, Universidad Autónoma de Barcelona, Bellaterra, 08193 Barcelona, Spain; ^3^Instituto de Ciencias Biomédicas, Universidad CEU-Cardenal Herrera, Avenida del Seminario s/n, Moncada, 46313 Valencia, Spain

## Abstract

After trauma brain injury, oxidative substances released to the medium provoke an enlargement of the initial lesion, increasing glial cell activation and, occasionally, an influx of immune cells into the central nervous system, developing the secondary damage. In response to these stimuli, microglia are activated to perform upregulation of intracellular enzymes and cell surface markers to propagate the immune response and phagocytosis of cellular debris. The phagocytosis of debris and dead cells is essential to limit the inflammatory reaction and potentially prevent extension of the damage to noninjured regions. Lipoic acid has been reported as a neuroprotectant by acting as an antioxidant and anti-inflammatory agent. Furthermore, angiogenic effect promoted by lipoic acid has been recently shown by our group as a crucial process for neural regeneration after brain injury. In this work, we focus our attention on the lipoic acid effect on astroglial and microglial response after brain injury.

## 1. Introduction

Traumatic brain injury (TBI) is a complex process involving a broad spectrum of symptoms and long-term consequences including disabilities [1]. As a consequence of the primary insult, many molecules from injured and dead cells induce microglial and astroglial activation [2] and disruption of the blood-brain barrier (BBB) [3]. In addition to the direct loss of tissue caused by the trauma, secondary mechanisms leading to additional tissue injury are important for outcome and therefore constitute important therapeutic targets [4].

Recently, interest has been focused on oxidative stress as a mechanism involved in the development of secondary brain damage [5]. After brain injury, a local increase in glial cell activation occurs and, occasionally, an influx of immune cells goes into the central nervous system (CNS). Accumulation of blood born immune cells at the side of the lesion is paralleled by activation of CNS-resident astrocytes and microglia, where they latter transform into phagocytic macrophages [6].

The use of lipoic acid (LA) on stroke and TBI animal models seems to be effective, restoring the BBB disruption and normalizing the astrocytic/microglial activation and glutathione (GSH) levels [7–9]. LA is well known as a natural cofactor for mitochondrial enzymes and is critical in breaking down fatty acids, which further enhance cellular energy efficiency. Recent findings obtained by our group show that LA works as a good neuroprotectant by acting as an antioxidant, increasing the antioxidant capacity of the tissue, decreasing the astroglial reaction, as well as the glial scar formation, promoting angiogenesis, and switching the regulation of several genes linked to cell survival and plasticity [10], but the microglial response is still unclear.

New controversies have also emerged such as the question of whether microglia are active or reactive players in neurodegenerative disease conditions. Some studies have been done to differentiate the inflammatory response from other biochemical processes in the development of secondary brain injuries and to explore what pathways in the inflammatory response mediate detrimental and/or protective effects following TBI [5]. It is now accepted that microglial cells can be acutely blood-derived in the adults under certain pathological conditions [11]. In that sense, microglial cells have the potential to develop into full-blown macrophages [12].

Since local microglia, astrocytes, and infiltrated macrophages are the main effectors of the innate immune response in the CNS [4], understanding the process of glial response is critical for formulating effective preventive and therapeutic strategies against brain injuries [13]. For that reason, the aim of the present work is to study the long-term astroglial and microglial response when LA treatment is administered after brain injury.

## 2. Experimental Procedures

### 2.1. Experimental Animal Models

Adult male Wistar rats (Harlan, Italy) weighing 250 ± 25 g were housed (two rats per box) one week before starting the experiment. Rats were housed in controlled conditions of temperature (20°C) and humidity (60%), under constant light-dark cycles of 12 hours. Handling and care of animals were done according to the *Real Decreto* 1201/05 and supervised and approved by the Committee of Ethics and Experimental Procedures of the Universidad CEU-Cardenal Herrera. Unnecessary stress or pain was avoided as possible.

#### 2.1.1. Surgery and Brain Cryoinjury

Rats were anesthetized with a mixture of ketamine (12 mg/kg), acepromazine (0.4 mg/kg), and fentanyl (0.02 mg/kg) that was injected intraperitoneally (i.p.). Once deeply anesthetized, rats were placed in a stereotaxic frame. The dorsal part of the skull was exposed and a craniotomy (2 mm of diameter) was drilled on stereotaxic coordinates anteroposteriorly, 0 mm from bregma, and laterally, 1.5 mm from medial line [14]. The brain cryoinjury was performed following the protocol described by Quintana et al. [15]. Thus, the cryoinjury (1 mm deep) was performed in the cerebral cortex by using a stainless steel probe (1 mm ø) previously frozen in liquid nitrogen. The frozen probe was maintained within the brain tissue for 20 sec. Finally, animals were sutured with a skin stapler (6.9 × 3.6 mm staples) and a mixture of buprenorphine (0.015 mg/kg) and metamizol (20 mg/kg) was i.p. administered after surgery.

Another group of rats were anesthetized, and the protocol described before was carried out (including the craniotomy), but rats were not cryoinjured. This group was considered as control group.

#### 2.1.2. Experimental Groups and LA Administration

Cryoinjured rats were randomly selected immediately after the surgery. One group (*n* = 12) received a daily dose (100 mg/kg i.p in NaCl 0.9%) of LA (Sigma Aldrich, Spain) for 7 days (CR + LA), starting the same day of surgery and the other group (*n* = 12) received the same volume of saline solution (NaCl 0.9% i.p.) for the same period of time (CR). Noncryoinjured rats (*n* = 12) received the same volume of saline (control).

#### 2.1.3. Sacrifice and Tissue Preparation

One pool of animals was housed for 15 days and then sacrificed by an i.p. overdose of pentobarbital (0.2 g/kg). Another pool was housed for 60 days and was sacrificed in the same way.

Once sacrificed, animals of 15 and 60 days (*n* = 6 for each group) were intracardially perfused with 100 mL of saline solution followed by 200 mL of 4% paraformaldehyde (PFA) in saline solution pH 7.5. Brains were removed and postfixed in the same fixative solution for 24 h at 4°C. Then, brains were cryoprotected by immersion in sucrose 30% in phosphate buffer saline (PBS) 0.01 M pH 7.1 solution for five days also at 4°C. Sections of 20 *μ*m thickness were serially obtained with a cryostat (Leica) and mounted in glass slides. Sections were stored at −80°C.

### 2.2. Cytological Study

#### 2.2.1. Hematoxylin-Eosine Staining

Haematoxylin-eosin staining was performed for histological characterization of the injured area by using brain sections from LA treated (CR + LA) and not treated animals (CR) at 60 days.

#### 2.2.2. Immunofluorescence

Sections were selected and washed three times with PBS 0.1 M for 5 min at room temperature, blocked for 2 h with 20% fetal bovine serum (FBS) in PBS-Triton 0.1%, and incubated at 4°C overnight with primary antibodies: anti-Collagen IV (1 : 200, Abcam, UK); anti-glial fibrillary acidic protein (GFAP) (1 : 500, Dako Cytomation, Denmark); and anti-CD68 (ED1) (1 : 200, AbD Serotec, UK).

Alexa Fluor 488 IgG (H + L) (1 : 200, Invitrogen, Spain) and Alexa Fluor 555 IgG (H + L) (1 : 200, Invitrogen, Spain) antibodies were used as secondary antibodies incubating for 2 h at room temperature in darkness. Afterwards, sections were mounted with DAPI Vectashield (Vector Laboratories, UK) and images were taken with a Leica Confocal Microscope.

#### 2.2.3. Immunocytochemical Staining

Sections were selected and rinsed for three times in PBS 0.1 M pH 7.5. In order to block unspecific binding sites and endogenous peroxidase, sections were incubated in darkness for 15 min in a solution of 3% H_2_O_2_-10% Methanol in PBS-Triton 0.1%. Afterwards, sections were incubated overnight at 4°C with specific primary antibody anti-rabbit Iba1 (Wako Chemicals, Germany) and 20% FBS in PBS-Triton 0.1% (1 : 200). Then sections were incubated with biotinylated goat anti-rabbit IgG antibody (1 : 200, Vector Lab, UK) in darkness for 2 h. After being washed three times in PBS, sections were incubated with avidin-biotin-peroxidase complex (Vector Lab, UK) for 1 h. Staining was developed with DAB (Vector Lab, UK) for time enough to make visible the marked cells. The reaction was stopped then by washing with distilled H_2_O and sections were washed with PBS to remove the excess. Finally, sections were dehydrated and coverslipped and images were taken with a Leica microscope.

### 2.3. Quantification and Statistical Analysis

Immunocytochemical and immunofluorescence images were quantified with ImageJ 1.44i for Mac. The quantifications were performed in the first 500 *μ*m from the injury limit. Six images were randomly taken of 4 sections of each animal (*n* = 6) and the number of cells was determinate by the nuclei presence and referred to the total area quantified (n° cells/mm^2^).

The number and length of the contacts were quantified with the program Leica LAS AF Lite for Windows Vista. Images were randomly taken of the first 500 *μ*m from the injury limit of different zones around the injured area. Regions of interest (ROIs) were identified when both colour markers (red and green) were overlapped. Each ROI was considered as a contact and the number of contacts (number of ROIs) referred to the total area measured (n° contacts/mm^2^). Total length of contacts (*μ*m) was quantified by adding the length of each individual ROI.

To perform the statistical analyses of the data, GraphPad Prism 4 for Mac was used. Statistical significance was assessed by one-way ANOVA followed by least significance differences test. Data are represented as mean ± standard deviation, and differences are considered significant at *P* < 0.05. Asterisks over the bars indicate statistically significant differences versus control.

## 3. Results

### 3.1. Histological Analysis of Brain Injury

**Figure 1:**
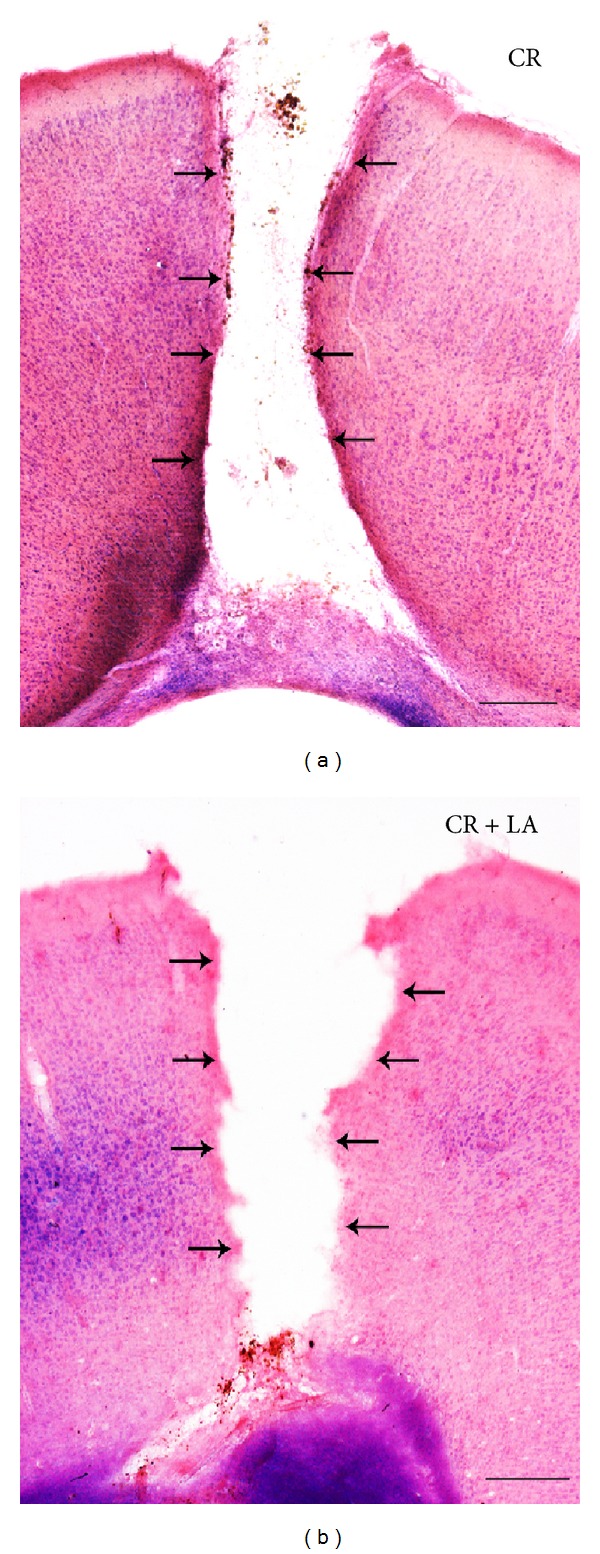
Hematoxylin-eosine staining of the injury area 60 days after the brain cryoinjury in untreated animals (a) and LA treated animals (b). Arrows indicate the edge of the injury. Scale bar: 500 *μ*m.

Haematoxylin-eosin staining of the brains was done, at 60 days, in order to evaluate the histology of the tissue. We found that untreated animals showed a perfectly delimited cystic cavity (Figure 1(a)), while LA treated animals showed an uneven edge of the injury (Figure 1(b)). These results are in agreement with those obtained previously by our group where we found that the LA treatment avoided the formation of the glial scar and produced growing of the neural tissue inside the cystic cavity [10].

### 3.2. Glial Reactivity after Brain Injury

**Figure 2:**
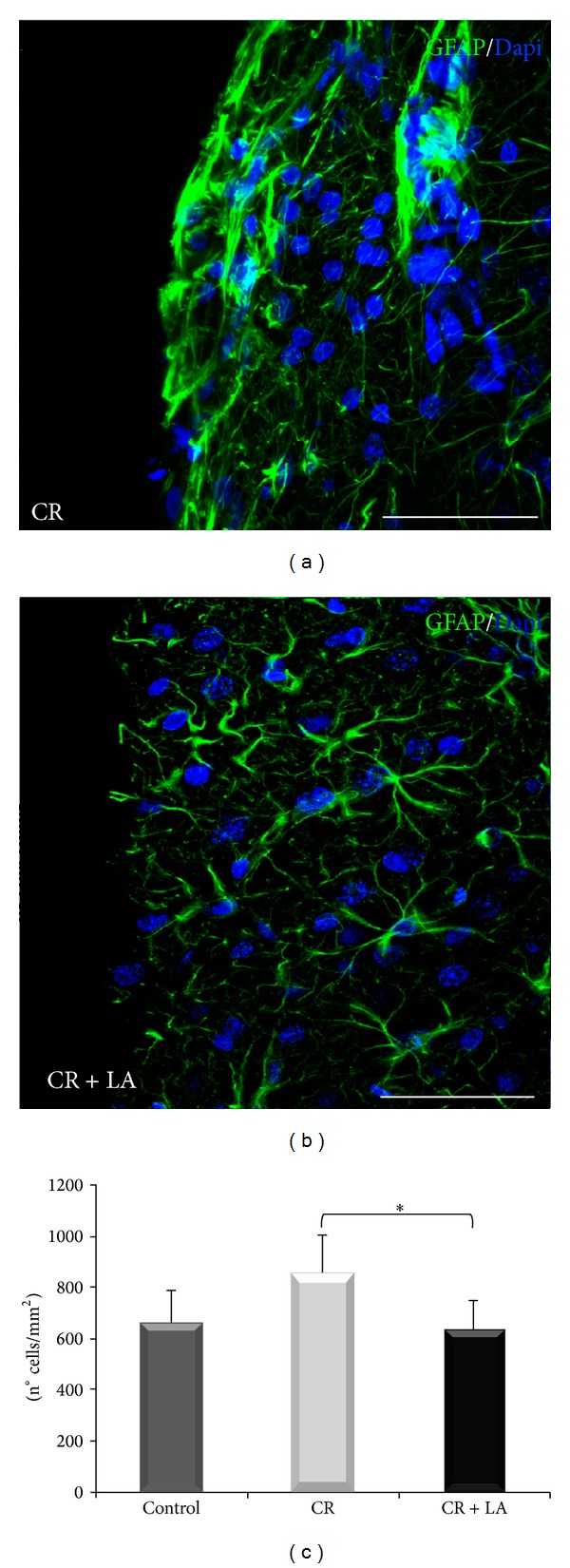
Immunofluorescence for GFAP (astrocytes in green) in nontreated animals (a) and LA treated animals (b), 60 days after the brain cryoinjury. Nuclei become evident with Dapi (blue). Quantification of astrocyte cell density (n° cells/mm^2^) (c) Scale bar: 50 *μ*m. *P < 0.05.

As has been seen before, our group has previously reported that the short-term effects of LA avoid the glial scar formation after the brain injury [10]. With the aim of evaluating the long-term astroglial reactivity after the brain injury, immunohistochemistry for GFAP (astrocytes) was carried out 60 days after the injury. In this case, untreated group (Figure 2(a)) still showed a marked astroglial scar in the injury limit while LA treated group (Figure 2(b)) presented a few astrocytes randomly distributed across de adjacent tissue. Moreover, a statistically significant decrease (*P* < 0.05) in the density of astroglial cells (Figure 2(c)) was shown in LA treated animals compared with nontreated animals.

### 3.3. Blood-Brain Barrier Formation after Brain Injury

**Figure 3:**
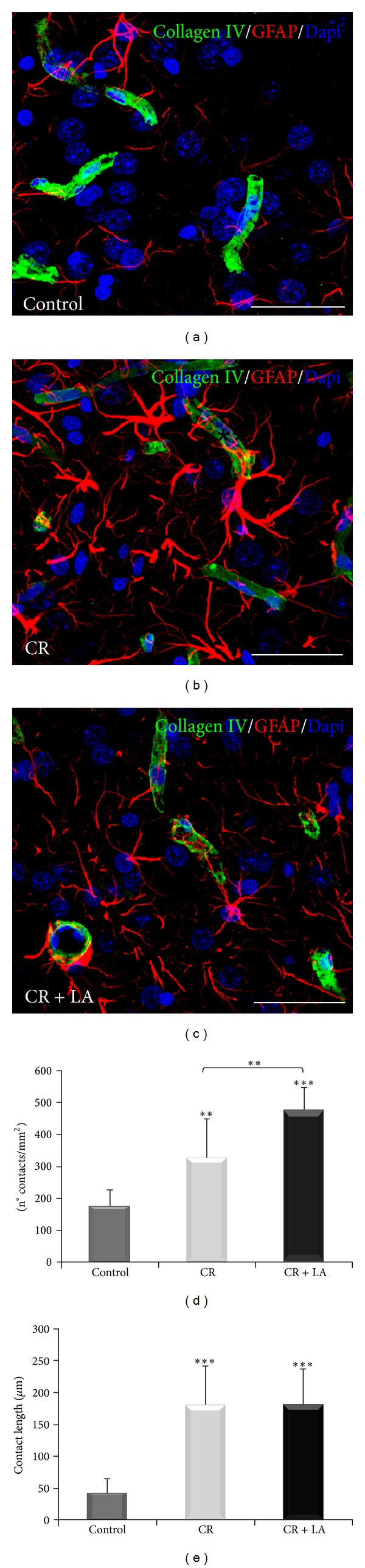
Immunofluorescence for GFAP (astrocytes in red) and Collagen IV (blood vessels in green) in control animals (a), nontreated animals (b), and LA treated animals (c) Nuclei become evident with Dapi (blue). Quantification of the density of contacts (n° contacts/mm^2^) established between astrocytes and blood vessels (d) and the total length of the contacts (*μ*m). (e) Scale bar: 20 *μ*m. **P < 0.01; ***P < 0.001.

It has been previously described that the BBB is affected after a TBI [3]. In order to evaluate the organization of the BBB, immunohistochemistry for Collagen IV (blood vessels) and GFAP (astrocytes) was done in control animals (Figure 3(a)), untreated animals (Figure 3(b)), and LA treated animals (Figure 3(c)) 15 days after the injury. The quantification of the number of contacts (n° contacts/mm^2^) established between astrocytes and blood vessels (Figure 3(d)) showed that both untreated and LA treated groups presented a statistical significant increase (*P* < 0.01 and *P* < 0.001, resp.) compared with control group. Moreover, LA treated group showed an increase (*P* < 0.01) compared with untreated group.

In addition, when the total length (*μ*m) of the contacts (Figure 3(e)) was measured, a statistically significant increase was observed in both experimental groups (CR and CR + LA) compared with control group (*P* < 0.001).

### 3.4. Microglial Response

**Figure 4:**
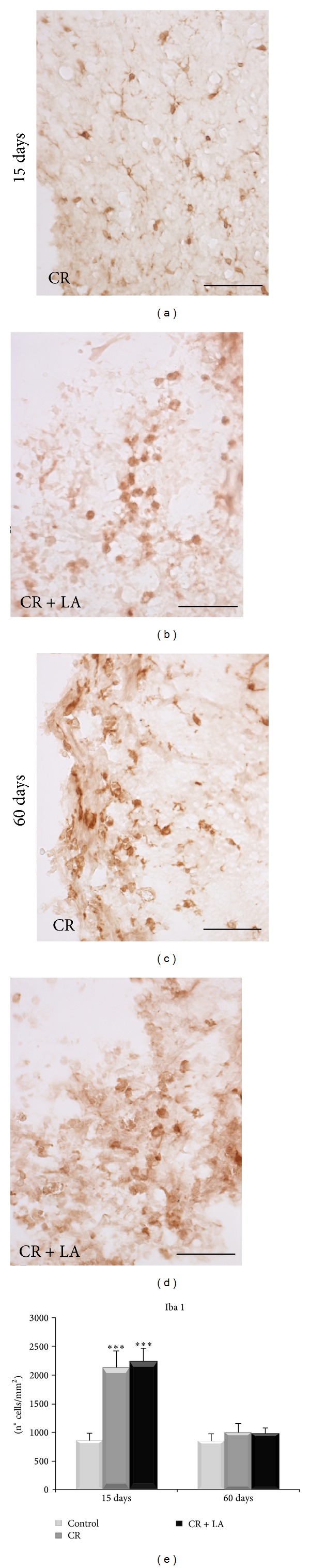
Immunocytochemistry for Iba1 (microglia) at 15 and 60 days in nontreated animals ((a) and (c), resp.) and LA treated animals ((b) and (d), resp.). Quantification of microglial cell density (n° cells/mm^2^) (e). Scale bar: 100 *μ*m. ***P < 0.001.

Microglial cells are the cell brain responsible of the immune response. Those were revealed by immunostaining with the marker Iba1 in order to see cell morphology (Figure 4). Images taken 15 days after the injury showed ramified microglia in untreated animals (Figure 4(a)) but amoeboid microglia cells were present in LA treated group (Figure 4(b)). On the other hand, images taken at 60 days revealed amoeboid microglial cells in both groups (Figures 4(c) and 4(d)). The quantification of the density of Iba1 positive cells (Figure 4(e)) showed a statistically significant increase in both experimental groups 15 days after the injury (*P* < 0.001) but showed no statistical differences after 60 days.

**Figure 5:**
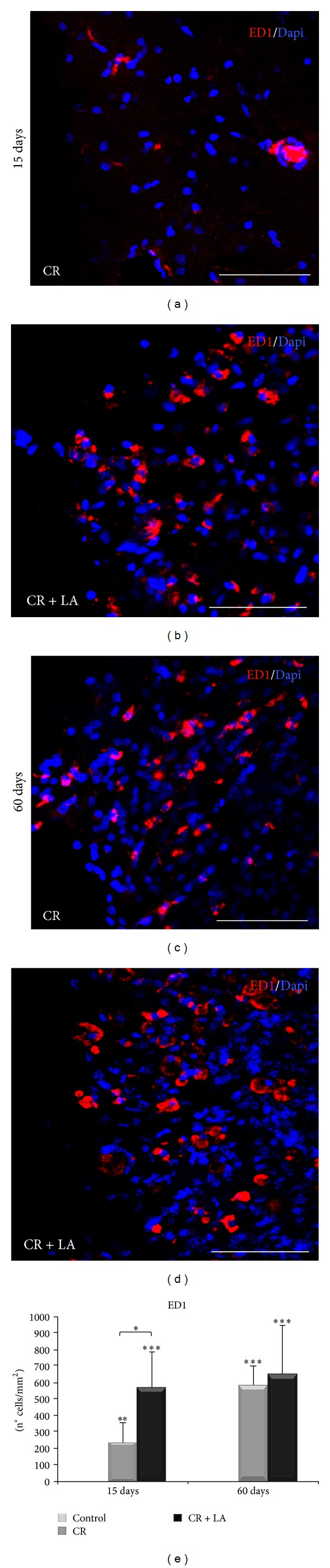
Immunofluorescence for ED1 marker (red) at 15 and 60 days in nontreated animals ((a) and (c), resp.) and LA treated animals ((b) and (d), resp.). Nuclei become evident with Dapi (blue). Quantification of cell density (n° cells/mm^2^) of activated microglia/macrophage (e) Scale bar: 200 *μ*m. **P < 0.05; **P < 0.01.

In addition, phagocytic cells were evaluated through immunohistochemistry for ED1 15 and 60 days after the injury (Figures 5(a)–5(d)). The quantification of the density of cells (n° cells/mm^2^) (Figure 5(e)) showed a statistically significant increase in both experimental groups 15 days after the injury (*P* < 0.01 and *P* < 0.001, resp.). Moreover, these phagocytic cells were statistically significantly increased in LA treated group compared with nontreated group (*P* < 0.05). Despite that, after 60 days, an increase in the density of ED1 positive cells was observed in untreated animals, reaching the same values as LA treated animals.

## 4. Discussion

After a TBI, inflammatory molecules from injured and dead cells are released to the extracellular medium, which elicits microglia and astroglia activation [2, 10].

Astrocytes respond to all forms of CNS insults through a process referred to as reactive astrogliosis [16]. It is well documented that the reactive astrocytes undergo hypertrophy; upregulate intermediate filaments composed of nestin, vimentin, and glial fibrillary protein (GFAP); and give rise to the glial scar [17]. Previous studies of our group showed that LA decreases the astroglial reactivity and avoids the glial scar formation 15 days after the brain injury [10]. In the present work we have shown that the glial scar still remains even 60 days after the injury while LA treatment avoids completely the scar formation in the long term and astroglial cells are randomly distributed across the adjacent tissue.

Moreover, it has been reported that the secondary damage detached from TBI contributes to the BBB disruption [7]. Herein, we have seen that LA promotes the formation of contacts between the endothelial cells and astrocytes, but we have shown that the length of contacts is increased after a TBI. Despite these findings were are not able to say if there is a restoration of the BBB; however some authors affirm that the use of LA on TBI and stroke animal models showed a restoration of the BBB disruption and normalization of the astrocytic/microglial activation and GSH levels [7–9].

Otherwise, activation of the immune system in the CNS has become increasingly recognized as a key component of the normal process of aging and also of the pathological onset and progression of many neurological disorders including TBI and neurodegenerative diseases [18]. Engulfment of apoptotic cells has traditionally been attributed to professional phagocytes, such as macrophages, microglia, and dendritic cells [16]. Recent advances in microglial biology have revealed that microglia may have important homeostatic functions [19]; in normal brain they are very active in surveillance of the normal neuronal environment and are the first cells to respond to any subtle changes [20]. It is well known that, under pathological conditions, microglia are rapidly activated and expanded in population to respond to the injury or stimulus. While ramified microglia are not in physical contact to each other, some authors have argued that their distribution within the brain allows them to “sense” their immediate surroundings. Individual cells could then respond to chemical or mechanical signals to activate the response to injury [21].

Herein, we have identified microglia morphology, to discriminate ramified microglia (resting) and amoeboid microglia (phagocytic). We have seen that microglia increased at 15 days and decreased at 60 days after the brain injury. However, only untreated group at 15 days showed ramified microglia. In addition, phagocytic cells were significantly increased in LA treated group at 15 days, while untreated group showed an increase at 60 days. In view of these results, we hypothesize that LA administration after brain injury avoids the glial scar formation, promoting the restoration of the tissue through early immune response developed by microglia.

Even though there is currently an open question about the role of the microglial cells, in general, it is evident that microglia play both detrimental and beneficial roles in brain injury, depending upon the time and severity of the inflammation. But on the other hand, there is also growing evidence showing that, under certain circumstances, microglia could be neuroprotective [22–24] and promote adult neurogenesis [25, 26]. However, it has been reported that innate immune response can also be beneficial in brain ischemia [27, 28]. Indeed, microglia have been shown to be neurosupportive by the uptake of glutamate [29], the removal of cell debris [30], and the engulfment of polymorphonuclear neutrophiles [31].

These findings, supported by our previous results, sustain LA treatment as a new regenerative strategy after brain injury and maybe other neurodegenerative diseases. However more studies should be done on the microglia response to clarify its role in this field.

## 5. Conclusion

Herein, we have reported that LA administration avoids the glial scar formation in the long term and promotes the BBB formation. Moreover, increases the microglial population early, but those decreases 60 days after the injury. In addition, early phagocytic cell appears close to the injury when LA is administered, while its absence delays the immune response activation and restoration of the tissue. All these findings lead us to better understanding of the immune response in the CNS after brain injury and represent a crucial step to the development of appropriate strategies against brain injury and other degenerative diseases.
